# Antimicrobial Resistance in *Enterococcus* spp. Isolated from Environmental Samples in an Area of Intensive Poultry Production

**DOI:** 10.3390/ijerph10031020

**Published:** 2013-03-12

**Authors:** Vesna Furtula, Charlene R. Jackson, Erin Gwenn Farrell, John B. Barrett, Lari M. Hiott, Patricia A. Chambers

**Affiliations:** 1 Environment Canada, Pacific Environment Science Centre, 2645 Dollarton Highway, North Vancouver, BC V7H 1B1, Canada; E-Mails: vesna.furtula@ec.gc.ca (V.F.); gwennfar@gmail.com (E.G.F.); Patricia.Chambers@ec.gc.ca (P.A.C.); 2 Bacterial Epidemiology and Antimicrobial Resistance Research Unit, USDA-ARS, Russell Research Center, 950 College Station Road, Athens, GA 30605, USA; E-Mails: benny.barrett@ars.usda.gov (J.B.B.); lari.hiott@ars.usda.gov (L.M.H.)

**Keywords:** Enterococci, antimicrobial, resistance, poultry litter, ground water, surface water, contamination

## Abstract

*Enterococcus* spp. from two poultry farms and proximate surface and ground water sites in an area of intensive poultry production were tested for resistance to 16 clinical antibiotics. Resistance patterns were compared to assess trends and possible correlations for specific antimicrobials and levels of resistance. Enterococci were detected at all 12 surface water sites and three of 28 ground water sites. Resistance to lincomycin, tetracycline, penicillin and ciprofloxacin in poultry litter isolates was high (80.3%, 65.3%, 61.1% and 49.6%, respectively). Resistance in the surface water to the same antibiotics was 87.1%, 24.1%, 7.6% and 12.9%, respectively. Overall, 86% of litter isolates, 58% of surface water isolates and 100% of ground water isolates were resistant to more than one antibiotic. Fifty-four different resistance patterns were recognised in isolates obtained from litter and environmental samples and several *E. faecium* and *E. faecalis* isolates from litter and environment samples shared the same resistance pattern. Multiple antibiotic resistant (MAR) indices calculated to assess health risks due to the presence of resistant enterococci suggested an increased presence of antibiotics in surface water, likely from poultry sources as no other wastewater contributions in the area were documented.

## 1. Introduction

Microbial contamination of water bodies due to municipal wastewater plants and animal operations is recognized as a growing issue. Wastewater and animal waste are reservoirs of antibiotic-resistance genes and multiple antibiotic-resistant (MAR, defined as resistance to at least two antimicrobials) pathogenic bacteria that pose a threat to human health [1,2,3]. Enterococci are a major colonizer of animal and human intestinal tracts and certain enterococcal strains may be source specific, making them suitable as a bacterial source tracking indicator [4,5]. Enterococci have been recognized as a leading cause of nosocomial infections, the majority of which are caused by *Enterococcus faecalis* and *Enterococcus faecium* [6,7], although other enterococcal species may also cause infections [8]. Enterococcal antimicrobial resistance has been observed in environmental isolates in many different studies [9,10,11]. Acquired resistance to a number of antibiotics, including vancomycin and aminoglycosides, presents a problem in treatment of enterococcal infections as well as posing a threat of resistance spreading into the environment via the transfer of antimicrobial resistance genes and some virulence factors from enterococci to pathogenic bacteria [12]. 

The Fraser Valley in British Columbia is the poultry capital of Canada, with poultry-related solid waste production exceeding 320,000 tonnes per year. Several studies have documented poultry litter as a potential reservoir for MAR bacteria [13,14,15]. Resistant bacteria and associated genes can persist over a long period of time in poultry litter and be subsequently released into the environment upon subsequent application of the litter as a fertilizer [13,16,17]. When poultry litter is used as a fertilizer or soil conditioner, multi-resistant bacteria can find their way into surface and ground waters via runoff or seepage, especially in areas where precipitation is plentiful [13,18]. Monitoring of antimicrobial resistance of fecal bacteria in surface water (mainly *E. coli* and *Enterococcus*) has been conducted throughout much of the World [11,19]. Although a few of these studies have investigated resistance of enterococcal species and their dissemination into surface water [20,21], to our knowledge environmental studies on occurrence of MAR bacteria in surface and ground water near poultry farms or farms using poultry litter as fertilizer have not been conducted. 

In this study, we investigated enterococci from different environments (poultry litter, surface and ground water) in areas of intensive poultry production to evaluate trends and correlations in specificity and levels of antibiotic resistance. MAR indices, the incidence of multiple-antibiotic resistant isolates among isolates from a sample, were calculated and applied to enterococci isolates to differentiate between low and high risk resistance bacterial contaminated sites. 

## 2. Experimental Section

### 2.1. Surface and Ground Water Sampling

Surface and ground water samples were collected from 12 and 28 sites, respectively, in the Abbotsford area of British Columbia, Canada, near poultry farms and berry farms that used poultry litter as fertilizer, as well as a reference site in a residential area in Port Moody, British Columbia [22]. Ground water sites were sampled in April, August, and December of 2009. Surface water samples were collected in December 2009 by submerging sterile 500 mL bottles approximately 50 cm below the water surface. For ground water samples, three full well volumes were purged from the piezometre using a submersible pump located close to the well screen. A minimum of three line volumes were purged from the sample tubing (low density polyethylene waterra tubing, dedicated for each well to prevent cross contamination) prior to sample collection using a Hydrolift pump. Both surface and ground water samples were transferred to 250 mL sterile polypropylene bottles containing sodium thiosulfate (10 mg/250 mL bottle) as provided by the laboratory. All water samples were placed on ice packs in coolers (~4 °C) and shipped to the laboratory where they were kept in a cold-room (≤4 °C). Samples were analyzed within 24 h of collection. 

Litter samples were collected from two different poultry farms, one broiler farm (where birds are reared for rapid growth and slaughtered for meat) and one layer farm (where hens are reared for egg production). Nine locations in four different barns of each farm type (broiler and layer) were sampled. Broiler farms were sampled on day 3 and day 35 of production (after application of new litter and introduction of birds). All samples were collected using gloves and sterile scoops and placed into sterile Falcon tubes. The samples were kept on ice until analysis, which was performed within 24 h of collection, except for samples from one layer barn where samples were frozen after collection and analyzed at a later date.

### 2.2. Isolation and Identification of Enterococci

Isolation of enterococci from water samples was performed using a membrane filtration technique. Samples (100 mL) were filtered through a 0.45 µm membrane sterile filter and incubated on mE agar for 48 h at 41 °C followed by incubation on Esculin Iron Agar (EIA) for 20 min at 41 °C as previously described [23]. Colonies that appeared pink to red with dark precipitation on EIA were verified using Biolog Microbial ID system in combination with the Biolog Gram Positive Aerobic Bacteria Database (Release 6.01, Biolog, Hayward, CA, USA). For poultry litter samples, 5–6 g of litter was weighed and dispensed into 10 mL of 0.85% sterile saline in a sterile 50 mL Falcon tube. The tube was vortexed on high for one minute and serial dilutions were plated on KF streptococcal agar (Difco, Detroit, MI, USA). Red or pink colonies on the KF agar were verified using Biolog Microbial ID system in combination with the Biolog Gram Positive Aerobic Bacteria Database (Release 6.01, Biolog, Hayward, CA, USA). Isolated colonies of confirmed *Enterococcus* were inoculated into 5 mL of tryptic soy broth containing 6.5% NaCl and incubated for 5–12 h at 35 °C; one mL of this culture was then combined with 325 µL of 80% glycerol and stored at −40 °C until further analysis. Isolates identified as the genus *Enterococcus* using Biolog were confirmed as *Enterococcus* and identified to species level using multiplex PCR [24]. 

### 2.3. Antimicrobial Resistance Testing

Minimum inhibitory concentrations (MIC, μg·mL^−1^) for enterococci were determined by broth microdilution using the Sensititre^TM^ semi-automated antimicrobial susceptibility system (Trek Diagnostic Systems, Inc., Cleveland, OH, USA) and the Sensititre^TM^ Gram-Positive Custom Plate CMV2AGPF. CLSI (Clinical and Laboratory Standards Institute, Wayne, PA, USA) antimicrobial resistance breakpoints were used whenever possible; however, no CLSI interpretive criteria have been defined for kanamycin and tylosin and only susceptible breakpoints (not resistant) have been established for daptomycin (≤4 μg·mL^−1^) and tigecycline (≤0.25 μg·mL^−1^). Breakpoints for daptomycin, kanamycin, lincomycin, tigecycline, and tylosin were those defined by the National Antimicrobial Resistance Monitoring System (NARMS) [25]. The Gram-Positive Custom Plate CMV2AGPF panel of 16 antimicrobials and breakpoints for classification as resistant used by the NARMS program and important in human medicine were as follows: chloramphenicol (≥32 μg·mL^−1^), ciprofloxacin (≥4 μg·mL^−1^), daptomycin (≥8 μg·mL^−1^), erythromycin (≥8 μg·mL^−1^), gentamicin (≥500 μg·mL^−1^), kanamycin (≥500 μg·mL^−1^), lincomycin (≥4 μg·mL^−1^), linezolid (≥8 μg·mL^−1^), nitrofurantoin (≥128 μg·mL^−1^), penicillin (≥16 μg·mL^−1^), streptomycin (≥1,000 μg·mL^−1^), Synercid^®^ (quinupristin/dalfopristin) (≥4 μg·mL^−1^), tetracycline (≥16 μg·mL^−1^), tigecycline (≥0.5 μg·mL^−1^), tylosin (≥32 μg·mL^−1^), and vancomycin (≥32 μg·mL^−1^). *Enterococcus faecalis* ATCC 29212, *E. faecalis* ATCC 51299, *Staphylococcus aureus* ATCC 29213 and *Escherichia coli* ATCC 25922 were used as quality controls for determination of MIC.

### 2.4. Data Analysis

Resistance results were interpreted according to CLSI guidelines when defined [26,27]. Categories of antimicrobial resistance were susceptible, intermediate and resistant according to NARMS classification [25].

### 2.5. MAR Index

The MAR index was calculated to compare the resistance level of isolates across different areas and sample types using the following equation [28]:

MAR_index_ = a/b**×**c (1)

where “a” represents number of antibiotics to which isolates were resistant, “b” represents the number of antibiotics to which isolates were exposed, and “c” represents the number of isolates per sample. 

## 3. Results and Discussion

### 3.1. Bacterial Recovery

Analysis of data from 12 surface water and 28 ground water sites in an area of intensive poultry farming showed that all surface water samples (n = 85) tested positive for *Enterococcus*, with counts ranging from 1 to 2,100 cfu/100 mL (Table 1). 

**Table 1:** Distribution of *Enterococcus* from surface water, ground water and poultry litter.

		No. (%) of samples containing:								
Sample	n	*E. faecalis*	*E. faecium*	*E. casseliflavus*	*E. durans*	*E. gallinarum*	*E. hirae*	*E. mundtii*	*E. raffinosus*	All other species
**Surface Water**										
S1	8	0	4 (50)	0	1 (12.5)	0	0	3 (37.5)	0	0
S2	5	0	3 (60)	2 (40)	0	0	0	0	0	0
S3	8	0	4 (50)	1 (12.5)	0	0	1 (12.5)	0	1 (12.5)	1 (12.5)
S4	10	5 (50)	1 (10)	0	0	0	1 (10)	3 (30)	0	0
S5	2	0	1 (50)	0	1 (50)	0	0	0	0	0
S6	10	4 (40)	4 (40)	0	1 (10)	1 (10)	0	0	0	0
S7	7	1 (14.3)	2 (28.6)	1 (14.3)	2 (28.6)	1 (14.3)	0	0	0	0
S8	10	10 (100)	0	0	0	0	0	0	0	0
S9	9	0	1 (11.1)	0	0	0	0	0	0	8 (88.9)
S10	2	1 (50)	1 (50)	0	0	0	0	0	0	0
S11	7	2 (28.6)	1 (14.3)	2 (28.6)	0	0	1 (14.3)	1 (14.3)	0	0
S12	7	0	0	2 (28.6)	1 (14.3)	0	0	4 (57.1)	0	0
**Total**	85	23 (27.1)	22 (25.9)	8 (9.4)	6 (7.1)	2 (2.4)	3 (3.5)	11 (12.9)	1 (1.2)	9
Ground Water										
BC-008	5	0	1 (20)	0	3 (60)	0	1 (20)	0	0	0
91-11	1	1 (100)	0	0	0	0	0	0	0	0
US-02	1	1 (100)	0	0	0	0	0	0	0	0
**Total**	7	2 (28.6)	1 (14.3)	0	3 (42.9)	0	1 (14.3)	0	0	0
Total Environment	92	25 (27.2)	23 (25)	8 (8.7)	9 (9.8)	2 (2.2)	4 (4.3)	11 (12)	1 (1.1)	9 (9.8)
Poultry Litter										
Layers	29	0	29 (100)	0	0	0	0	0	0	0
Broilers (day 3)	105	30 (28.6)	16 (15.2)	1 (0.95)	1 (0.95)	28 (26.7)	27 (25.7)	0	0	2 (1.9)
Broilers (day 35)	29	6 (20.7)	21 (72.4)	0	1 (3.4)	0	1 (3.4)	0	0	0
**Total**	163	36 (22.1)	66 (40.5)	1 (0.6)	2 (1.2)	28 (17.2)	28 (17.2)	0	0	2 (1.2)

By comparison, the reference site sampled on all three occasions did not test positive for *Enterococcus*. Enterococci were detected in three of the 28 ground water sites (n = 92 water samples; Table 1) with bacterial counts ranging from 1 to 5 cfu/100 mL. Although there are no regulations for enterococci in surface or ground water in Canada, several locations did not meet the mandatory European Union standards of 400 cfu/100 mL for inland waters and 200 cfu/100 mL for coastal or transitionary waters [29]. 

Enterococci have long been recognized as an indicator of fecal contamination; however, there are few studies about their resistance and distribution in surface water [20,21] and to our knowledge, no such studies for ground water. In our study, seven enterococci were isolated from ground water samples from three sites, 85 were isolated from 12 surface water sites, and 163 were isolated from poultry litter, for a total of 255 isolates. All presumptive enterococcal strains were confirmed and classified; five isolates originally found in the samples could not be resuscitated on the standard media used in this study and were not included in the further experiments. Among the surface water samples, out of 23 possible *Enterococcus* species [24], nine species (including all other species) were detected and their percentages differed among locations and environmental compartments (Table 1). *E. faecalis* and *E. faecium* (characteristic of the digestive tract of human and warm-blooded animals) were the predominant species in surface water (27% and 26%, respectively), consistent with previous reports [2,9,30]. *E. casseliflavus, E. gallinarum, E. hirae* and *E. durans* were isolated from the poultry litter samples in this study, which is consistent with the literature, in that they are generally regarded as animal-derived strains, found in the gastrointestinal tract of poultry [31,32]. Our observation of their occurrence in surface and ground water samples may be indicative of contamination from the poultry farms of these environmental samples.

### 3.2. Antimicrobial Resistance

Antimicrobial resistance is a major global health concern, leading to development of monitoring programs such as the National Antimicrobial Resistance Monitoring Systems (NARMS) [33] in the USA and the European Antimicrobial Resistance Surveillance System (EARSS) [34] in Europe. Two hundred and fifty enterococcal isolates were tested for antimicrobial susceptibility; all 250 isolates were found to be susceptible to linezolid and tigecycline regardless of origin (litter, surface or ground water) and all isolates except two (both from broiler litter) were susceptible to gentamicin. All isolates except three (one from surface water site S6 and two from poultry litter) were resistant to at least one of the 16 clinical antibiotics tested and five isolates from broiler litter were resistant to nine antibiotics. Low resistance to gentamicin is consistent with previous studies [35,36,37]. Only one isolate (0.39%) was resistant to chloramphenicol (surface water sample from location S6). 

#### 3.2.1. Litter Samples

**Figure 1:**
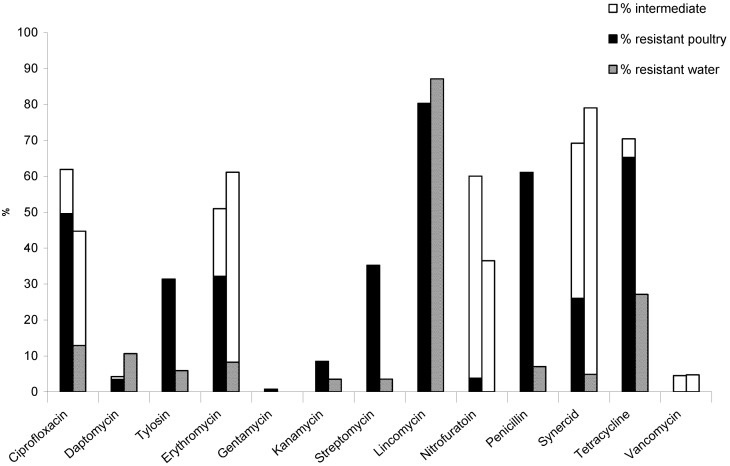
Percent antibiotic resistance for enterococci. Enterococci isolated from poultry litter and environmental water samples were tested against a panel of 16 antimicrobials. Percent resistant enterococci from poultry litter (solid bar) and water (hatched bar) are shown for each antimicrobial; intermediate resistant isolates for each source are shown in the open bars. All isolates were susceptible to linezolid and tigecycline; only one isolate (*E. faecalis* from water) was resistant to chloramphenicol (data not shown).

Enterococci isolates from litter from both farms showed high resistance (>50% resistant) to lincomycin (80.3%), tetracycline (65.3%), and penicillin (61.1%) (Figure 1). In contrast, resistance to ciprofloxacin (49.6%), streptomycin (35.2%), erythromycin (32.2%), tylosin (31.4%), and Synercid^®^ (26.0%) was classified as medium (25–50% resistance), whereas resistance to kanamycin (8.5%), nitrofurantoin (3.8%), daptomycin (3.5%) and gentamicin (0.8%) was low (<25% resistant) (Figure 1). Similar resistance values were observed for ciprofloxacin, nitrofurantoin and penicillin for litter from layers and broilers day 35 (both sampled from aged litter). High resistance to lincomycin and tetracycline by enterococci was observed for both the day 3 and day 35 broiler litter samples, whereas enterococci from layers had much lower resistance to these two antibiotics. This could be due to larger quantities of antimicrobial agents and growth promoters used in broiler compared to egg-laying husbandry. A previous study of antimicrobial resistance of two enterococcal species, *E. faecium* and *E. faecalis,* isolated from poultry litter likewise reported much lower resistance to erythromycin, ciprofloxacin and streptomycin for *E. faecium* from layers compared to broilers [38]. Our data also showed much lower resistance to all antibiotics (except daptomycin) for *E. faecium* isolated from layer compared to broiler litter. Resistance levels in *E. faecium* and *E. faecalis* from broilers were similar to results in a previous study conducted in Belgium [35]. 

Our results as well as two other studies [35,38] confirmed high resistance of enterococci from poultry litter to lincosamides (lincomycin in this study). Those studies also reported high levels of resistance to macrolides whereas we reported medium levels of resistance to erythromycin and tylosin. Taken together, these results suggest similarities around the globe. Interestingly, high resistance to tetracycline (75.6%), erythromycin (56.8%) and ciprofloxacin (41.9%) were reported in a previous study of *Enterococcus* isolated from poultry intestines immediately after slaughtering [39]. However, analysis of changes in prevalence and patterns of antimicrobial resistance among *Enterococcus* spp. isolated from growing broilers and their feces compared to a control group indicated that antimicrobials were not necessarily the cause of increased resistance to ampicillin, tetracycline, erythromycin and nitrofurantoin [40]. Rather, strains present in feed and farmhouse environments may colonize broiler intestines and cause shifts in the prevalence of resistance. Regardless of the underlying cause of resistance, it is critical that management practices be implemented such that resistant enterococci found in litter do not make their way into the environment and cause changes in the resistance patterns of environmental bacteria.

Only 4 (14%) of the litter isolates from the layer farm were resistant to a single antibiotic, and 6 (21%) were resistant to seven different antibiotics. All isolates from the broiler farm were MAR (resistant to at least two antibiotics). More recent studies are in agreement with our results, reporting higher levels of resistance to Synercid^®^ and tylosin [41,42].

#### 3.2.2. Water Samples

Forty-one percent of enterococci surface water isolates (n = 36) were resistant to one antibiotic and 59% were MAR. Resistance of enterococci from surface water samples to lincomycin was high (87.1%); resistance to tetracycline (27.1%) was medium, while low resistance was observed for ciprofloxacin (12.9%), Synercid^®^ (15.7%) daptomycin (10.6%), erythromycin (8.2%), tylosin (5.9%), streptomycin (3.5%), penicillin (7.6%), and kanamycin (3.5%). In addition to these isolates with full resistance, there were a number of isolates with intermediate resistance to ciprofloxacin (31.8%), erythromycin (52.9%), Synercid^®^ (74.2%), nitrofurantoin (36.5%) and vancomycin (1.2%). Previous studies reported little to no vancomycin resistance in poultry production environments [37,42,43,44]. Use of avoparcin is associated with emergence of vancomycin resistant *Enterococcus* (VRE), but since avoparcin has never been used in poultry production in Canada or the USA, our findings are in accordance with other studies [36,43]. The presence of intermediate resistant isolates could indicate a trend toward full resistance and in some studies intermediate is counted as resistant [30]. If that approach had been taken in this study, the percentage of resistant isolates would increase significantly as many isolates investigated in this study showed intermediate resistance (Figure 1). All ground water isolates were MAR, resistant to at least two antibiotics and as many as four antibiotics.

When resistance in the environmental isolates is examined by species (Table 2), all *E. faecium* and *E. faecalis* isolates (24.5% and 26.6% of total samples, respectively) were susceptible to daptomycin and vancomycin. Lincomycin and tetracycline resistance levels were noticeably higher in *E. faecalis* than *E. faecium. E. faecium* had higher levels of ciprofloxacin and penicillin resistance (Table 2). It should be noted that *E. faecalis* is intrinsically resistant to Synercid^®^, thus those levels cannot be compared between the two species. *E. hirae and E. durans* from water samples and poultry samples were resistant to lincomycin, tetracycline, tylosin, erythromycin and nitrofurantoin (Table 2) which could be indicative of the same origin. Enterococci isolated from untreated waters for human consumption in Portugal have also shown resistance to ciprofloxacin, erythromycin and tetracyclines, implying that resistance to these antibiotics may be a widespread issue [9].

**Table 2:** Antimicrobial resistance profiles of enterococci from surface water and poultry litter samples from both broiler and layers barns.

Antimicrobial	Break-point (µg/mL)	Source	No. (%) of isolates resistant					
			*E. faecalis* (n = 57)	*E. faecium* (n = 88)	*E. gallinarum* (n = 30)	*E. hirae* (n = 32)	*E. durans* (n = 11)	All other species (n = 32)
Chloramphenicol	≥32	Water	1 (1.8)	0	0	0	0	0
		Layers		0				
		Broilers	0	0	0	0	0	0
Ciprofloxacin	≥4	Water	1 (1.8)	11 (12.5)	0	0	0	1
		Layers		22 (25)				
		Broilers	0	25 (28.4)	2 (6.7)	1 (3.1)	0	0
Daptomycin	≥8	Water	0	0	0	1 (3.1)	0	9
		Layers		2 (2.3)				
		Broilers	0	0	0	0	0	0
Tylosin	≥32	Water	2 (3.5)	0	0	1 (3.1)	3 (27.2)	0
		Layers		6 (6.8)				
		Broilers	17 (29.8)	6 (6.8)	25 (83.3)	2 (6.3)	2 (18.2)	3
Erythromycin	≥8	Water	2 (3.5)	1 (1.1)	0	1 (3.1)	3 (27.2)	1
		Layers		6 (6.8)				
		Broilers	17 (29.8)	8 (9.1)	25 (83.3)	2 (6.3)	2 (18.2)	3
Kanamycin	≥1,024	Water	1 (1.8)	1 (1.1)	0	1 (3.1)	0	0
		Layers		1 (1.1)				
		Broilers	4 (7.0)	6 (6.8)	5 (16.7)	2 (6.3)	0	0
Streptomycin	>1,000	Water	1 (1.8)	1 (1.1)	0	0	3 (27.2)	0
		Layers		8 (9.1)				
		Broilers	8 (14.0)	22 (25)	20 (66.7)	1 (3.1)	2 (18.2)	1
Lincomycin	>1,000	Water	24 (42.1)	13 (14.8)	2 (6.7)	4 (12.5)	9 (81.8)	29
		Layers		18 (20.5)				
		Broilers	32 (56.1)	34 (38.6)		28 (87.5)	2 (18.2)	3
Nitrofurantoin	≥128	Water	0	2 (2.3)	0	1 (3.1)	4 (36.4)	0
		Layers		0				
		Broilers	0	4 (4.5)	0	4 (12.5)	1 (0.9)	0
Penicillin	≥16	Water	0	5 (5.7)	0	1 (3.1)	0	0
		Layers		23 (26.1)				
		Broilers	0	31 (35.2)	0	24 (75)	0	0
Synercid^®^	≥4	Water	24 (42.1)	1 (1.1)	0	1 (3.1)	0	1
		Layers		2 (2.3)				
		Broilers	32 (56.1)	19 (21.6)	3 (10)	1 (3.1)	2 (18.2)	1
Tetracycline	≥16	Water	13 (22.8)	7 (8.0)	0	1 (3.1)	5 (45.5)	1
		Layers		13 (14.8)				
		Broilers	22 (38.6)	31 (35.2)	27 (90)	27 (84.4)	2 (18.2)	2
Gentamicin	≥500	Water	0	0	0	0	0	0
		Layers		0				
		Broilers	0	0	0	2 (6.3)	0	0

#### 3.2.3. Resistance Patterns

A total of 54 resistance patterns for litter and environmental enterococci isolates were observed (Table 3). Only 17% of isolates were resistant to one of the 16 antibiotics tested. Resistance to a single antibiotic may not be a meaningful measure for study comparisons because the same isolates may be resistant to other antibiotics not tested. 

**Table 3:** Antibiotic resistance patterns for *Enterococcus* spp. in litter and water.

No. antimicrobials	Resistance pattern ^a^	Species (No. isolates)	Source	
			Litter	Environment
9	Lin Tet Pen Tyl Cip Str Syn Kan Nit	*E. faecium* (1)	1	
	Lin Tet Pen Tyl Ery Cip Str Syn Kan Ni	*E. faecium* (4)	4	
8	Lin Tet Pen Tyl Ery Str Cip Syn	*E. faecium* (1)	1	
	Lin Tet Tyl Ery Str Kan Chl	*E. faecalis* (1)		1
7	Lin Tet Pen Ery Str Syn Cip	*E. faecium* (1)	1	
	Lin Tet Tyl Ery Str Syn Kan	*E. faecalis* (4)	4	
		*E. gallinarum* (3)	3	
	Lin Tet Pen Cip Str Syn Nit	*E. hirae* (1)	1	
	Lin Tet Tyl Ery Str Syn Nit	*E. durans* (1)	1	
	Lin Pen Tet Tyl Ery Cip Str	*E. faecium* (3)	3	
	Lin Pen Tet Tyl Ery Str Syn	*E. faecium* (1)	1	
	Lin Pet Tet Tyl Ery Str Kan	*E. faecium* (1)	1	
	Lin Pen Tyl Cip Ery Str Syn	*E. faecium* (1)	1	
6	Lin Tet Pen Cip Syn Lin	*E. faecium* (9)	9	
	Lin Tet Tyl Str Syn Ery	*E. faecalis* (1)	1	
	Lin Tet Tyl Ery Str Kan	*E. gallinarum* (2)	2	
		*E. durans* (1)	1	
5	Lin Tet Pen Cip Str	*E. faecium* (4)	4	
	Lin Tet Tyl Ery Syn	*E. faecalis* (13)	12	1
	Lin Tet Tyl Ery Str	*E. gallinarum* (15)	15	
		*E. durans* (2)		2
	Lin Tet Pen Gen Kan	*E. hirae* (2)	2	
	Lin Tet Pen Cip Dap	*E. faecium* (2)	2	
4	Lin Tet Pen Str	*E. faecium* (2)	2	
	Lin Tet Pen Syn	*E. faecium* (2)	2	
	Lin Tet Pen Cip	*E. faecium* (3)	3	
	Lin Tet Ery Tyl	*E. gallinarum* (5)	5	
		*E. hirae* (2)	2	
		*E. durans* (1)		1
		*E.* species (1)	1	
	Lin Tet Str Tet	*E. faecium* (1)		1
	Lin Tet Ery Kan	*E. faecium* (1)		1
	Lin Tet Pen Nit	*E. hirae* (3)	3	
	Lin Pen Str Cip	*E. faecium* (1)	1	
3	Pen Cip Nit	*E. faecium* (1)	1	
	Tet Pen Cip	*E. faecium* (4)	3	1
	Lin Tet Ery	*E. faecium* (1)	1	
		*E. raffinosus* (1)		1
	Lin Tet Syn	*E. faecalis* (17)	5	12
	Lin Tet Str	*E. durans* (1)		1
	Lin Str Syn	*E. faecalis* (3)	3	
	Lin Tet Cip	*E. faecium* (1)		1
		*E. gallinarum* (1)	1	
	Lin Tet Pen	*E. faecium* (2)	1	1
		*E. hirae* (18)	18	
	Lin Tyl Ery	*E.* species (1)	1	
	Lin Syn Cip	*E. faecalis* (1)		1
	Lin Pen Cip	*E. faecium* (6)	6	
	Tet Cip Str	*E. faecium* (1)	1	
2	Lin Tet	*E. faecium* (4)	4	
		*E. gallinarum* (1)	1	
		*E. hirae* (1)	1	
		*E. durans* (1)		1
	Lin Nit	*E. faecium* (1)	1	
	Lin Syn	*E. faecalis* (16)	7	9
		*E. hirae* (1)		1
		*E.* species (1)		1
	Pen Tet	*E. faecium* (2)		2
		*E. gallinarum* (1)	1	
	Pen Cip	*E. faecium* (6)	3	3
		*E. casseliflavus* (1)		1
	Lin Dap	*E. hirae* (1)		1
		*E. mundtii* (11)		11
1	Pen	*E. faecium* (2)	2	
	Lin	*E. faecium* (5)		5
		*E. faecalis* (1)		1
		*E. gallinarum* (2)		2
		*E. hirae* (2)	1	1
		*E. durans* (4)		4
		*E. casseliflavus* (7)		7
		*E.* species (8)		8
		*E. mundtii* (1)		1
	Cip	*E. faecium* (10)	3	7
	Tet	*E. faecium* (1)		1
Total			157	93

^a^ Cip = Ciprofloxacin, Chl = Chloramphenicol, Dap = Daptomycin, Ery = Erythromycin, Gen = Gentamicin, Kan = Kanamycin, Lin = Lincomycin, Nit = Nitrofurantoin, Pen = Penicillin, Str = Streptomycin, Syn = Synercid^®^, Tet = Tetracycline, Tyl = Tylosin.

For example, a previous study of bacitracin resistance by enterococci isolates from chicken ceca or feces showed that all samples were resistant to at least two different classes of antibiotics, and bacitracin resistance was present in all patterns [43]. Bacitracin was excluded from USDA NARMS plates because virtually all enterococci, regardless of species, are resistant to that antimicrobial. Another study in France observed that three of 16 antibiotics tested (largely different than those tested in this study) accounted for 96% of antimicrobial resistance present in *E. coli* isolated from rivers [19]. This confirms that the choice of antibiotics determines the prevalence of AR, making comparison among studies difficult. 

Several *E. faecium* and *E. faecalis* isolates from litter and the environment had the same resistance pattern (ciprofloxacin, ciprofloxacin/penicillin, lincomycin/tetracycline/Synercid^®^, lincomycin/tetra-cycline/tylosin/erythromycin/Synercid^®^; Table 3). The resistance patterns of the isolates reflect the antibiotic use in poultry production in the area; for example, lincomycin, tetracycline, penicillin, and tylosin use was reported previously [43]. Resistance to erythromycin and tetracycline was also reported in Denmark for *E. faecium* and *E. faecalis* in broilers [45]. Unlike many other coliforms (such as *E. coli*), intestinal enterococci species antimicrobial resistance properties differ notably between humans and different species of animals, resulting in specific patterns which could be used to differentiate of contamination sources.

### 3.3. MAR Indices

To assess the relative prevalence of resistant enterococci in the environment, MAR indices were calculated and compared with those previously published (Table 4). For all surface water sites, MAR indices were between 0.06 and 0.19; for litter samples, the average MAR index was 0.27 ± 0.07. In a study of fecal discharge to the Seine River, *Enterococcus* MAR indices were found to be 0.24 for a point source (i.e., hospital wastewaters), indicating high antibiotic use, compared to values of 0.078 for an agricultural non-point source and 0.168 for the river itself [19]. Panda *et al.* [11] monitored MAR pathogens in the Bay of Bengal, India and reported a high MAR index of 0.083 although it is not clear for which bacteria it was calculated, which may be important based on data reported for *E. coli* [19,28]. MAR indices calculated in this study for litter (0.27) were approximately double the water values. Although the introduction of resistant enterococci into the environment from farm run-off would be diluted during passage to proximate surface waters, the relatively high MAR indices at some surface water sites likely indicates inputs from poultry operations as no other wastewater sources in the area were observed. Because there are no criteria for MAR index for enterococci, it is difficult to assess human health risks due to presence of resistant enterococci in the water. Based on comparison of MAR indices from *E. coli* isolates from a variety of sources, Krumperman [28] suggested a MAR index of 0.200 to differentiate between low and high-risk contamination, although he acknowledged that this value was arbitrary. Although our results comparing litter and surface water MAR indices suggest poultry contamination of the environment, the risk posed by this contamination may be low given all surface water MAR values were <0.2. Further detailed studies of MAR indices for enterococci are needed for risk assessment.

**Table 4:** AR, MAR and MAR indices for enterococci isolates.

		AR	MAR		MAR index
		≥1 (%)	≥2 (%)	≥5 (%)	
**Surface water**					
S1		100	63	13	0.141
S2		100	40	0	0.071
S3		100	75	0	0.133
S4		100	80	10	0.138
S6		90	40	10	0.131
S7		100	29	29	0.125
S8		0	100	0	0.188
S9		100	0	0	0.063
S11		100	71	0	0.107
S12		100	71	0	0.107
**Groundwater**					
		0	5	0	0.188
**Poultry farms**					
**Layers**		97	83	28	0.218
**Broilers (day3)**		100	98	44	0.248
**Broilers (day 35)**		100	100	83	0.358

AR = resistance to one antibiotic; MAR = resistance to at least two antibiotics.

## 4. Conclusions

This study confirmed the presence of resistant enterococci species in the environment, specifically surface and ground water. The majority of isolates were MAR and some water isolates exhibited the same resistance pattern as isolates from poultry litter. Although these antibiotics are not used in poultry production, resistance to lincomycin, tetracycline, penicillin, and ciprofloxacin in surface water and litter was observed. These resistances may have resulted from cross-resistance to other antibiotics in the same class which are used in poultry production. Cross-resistance to antibiotics in enterococci may limit antibiotic efficacy in human medicine. MAR indices calculated for surface water samples suggest increased presence of antibiotic resistant enterococci in the surface water tested. Results from this study could be beneficial for improvement of best management practices in the area.
